# Rural health centres, communities and malaria case detection in Zambia using mobile telephones: a means to detect potential reservoirs of infection in unstable transmission conditions

**DOI:** 10.1186/1475-2875-9-96

**Published:** 2010-04-15

**Authors:** Aniset Kamanga, Petros Moono, Gillian Stresman, Sungano Mharakurwa, Clive Shiff

**Affiliations:** 1 The Malaria Institute at Macha, P O Choma, Zambia; 2 Department of Molecular Microbiology and Immunology, Johns Hopkins Bloomberg School of Public Health, Baltimore, MD, USA

## Abstract

**Background:**

Effective malaria control depends on timely acquisition of information on new cases, their location and their frequency so as to deploy supplies, plan interventions or focus attention on specific locations appropriately to intervene and prevent an upsurge in transmission. The process is known as active case detection, but because the information is time sensitive, it is difficult to carry out. In Zambia, the rural health services are operating effectively and for the most part are provided with adequate supplies of rapid diagnostic tests (RDT) as well as effective drugs for the diagnosis and treatment of malaria. The tests are administered to all prior to treatment and appropriate records are kept. Data are obtained in a timely manner and distribution of this information is important for the effective management of malaria control operations. The work reported here involves combining the process of positive diagnoses in rural health centres (passive case detection) to help detect potential outbreaks of malaria and target interventions to foci where parasite reservoirs are likely to occur.

**Methods:**

Twelve rural health centres in the Choma and Namwala Districts were recruited to send weekly information of rapid malaria tests used and number of positive diagnoses to the Malaria Institute at Macha using mobile telephone SMS. Data were entered in excel, expressed as number of cases per rural health centre and distributed weekly to interested parties.

**Results:**

These data from each of the health centres which were mapped using geographical positioning system (GPS) coordinates were used in a time sensitive manner to plot the patterns of malaria case detection in the vicinity of each location. The data were passed on to the appropriate authorities. The seasonal pattern of malaria transmission associated with local ecological conditions can be seen in the distribution of cases diagnosed.

**Conclusions:**

Adequate supplies of RDT are essential in health centres and the system can be expanded throughout the country to support strategic targeting of interventions by the National Malaria Control Programme. Participation by the health centre staff was excellent.

## Background

Malaria control as a national programme involves careful management on a day-to-day basis if it is to be sustained and effective. Such management requires acquisition of data on the ebb and flow of transmission across the numerous ecosystems and throughout the seasonal variations that occur in an endemic area. In the absence of a stream of accurate and timely data on occurrence of malaria cases, the control processes may go adrift [1]. Unfortunately, data collection in many parts of the developing world is difficult to obtain and disseminate, hence administrators put reliance on annual or periodic assessments made either on an *ad hoc *basis or as occasional cross-sectional studies [2]. The difficulty with information at such a level is that it does not deal with malaria at the local level. Transmission is very much dependent on local conditions, both geographical and seasonal and frequently it is only on the micro geographical scale that precise, time sensitive information about changes in transmission can alert administrators appropriately. Where transmission is unstable, malaria cases spread frequently from defined foci. The question arises, can a system be put in place that involves local health facilities, and collects information about case occurrences in a timely and efficient manner to help identify such foci?

In Zambia, the local health system consists of widely dispersed rural health centres which operate under the authority of the District Medical Director (DMD). These centres serve the public and collect information of illness and illness outbreaks on a timely basis. In some instances, the validity of data collected at rural health establishments has been called into question [3]; however this does not preclude using the data in situations where one has confidence in the health system and personnel involved, as is the case now in Zambia [4]. Normally these centres are supplied with malaria rapid diagnostic tests (RDT), anti-malarial drugs as well as other health related supplies. Stockouts may occur, but for the most part of this study period adequate supplies of RDT and appropriate drugs were available. In the case of a suspected malaria case, Ministry of Health protocol requires the infection to be confirmed by the RDT prior to treatment. This specific information is recorded and submitted periodically to the District Medical Director (DMD). However, the information represents diagnoses *in time *and as such is valuable information that could alert public health authorities to the existence of transmission episodes and potential clustering of malaria cases. Such data, if appropriately available, can be analysed locally and is important to predict outbreaks of malaria and can be used to target specific interventions to restrict a spread of the disease. The research described here has taken advantage of the Zambian rural health system and through the use of personal mobile telephones, demonstrates how the data can be processed in a manner useful to manage the malaria interventions in current use.

Two examples of modern technology have revolutionized the rural health centre in Africa, the RDT and the cell phone. In the case of malaria in the past, records of clinical diagnoses have been unreliable because of the lack of specificity [5] but the advent of the malaria rapid diagnostic test has revolutionized the role played by these health centres. A clear diagnostic result can be obtained rapidly and with acceptable accuracy [6]. In Zambia, the Ministry of Health through the National Malaria Control Centre, maintains data on the quality of RDT approved for use and purchases stocks under tender from responsible sources. The cell phone places the RHC in direct contact with the Ministry of Health and other stakeholders. Thus information collected is available, timely and of local importance. This report demonstrates how 12 of these centres and their staff were recruited to serve as a data collection resource for malaria surveillance and show the value of information collected for epidemiological support for the National Malaria Control Centre and the Ministry of Health.

## Methods

### Locality and logistics

**Figure 1:**
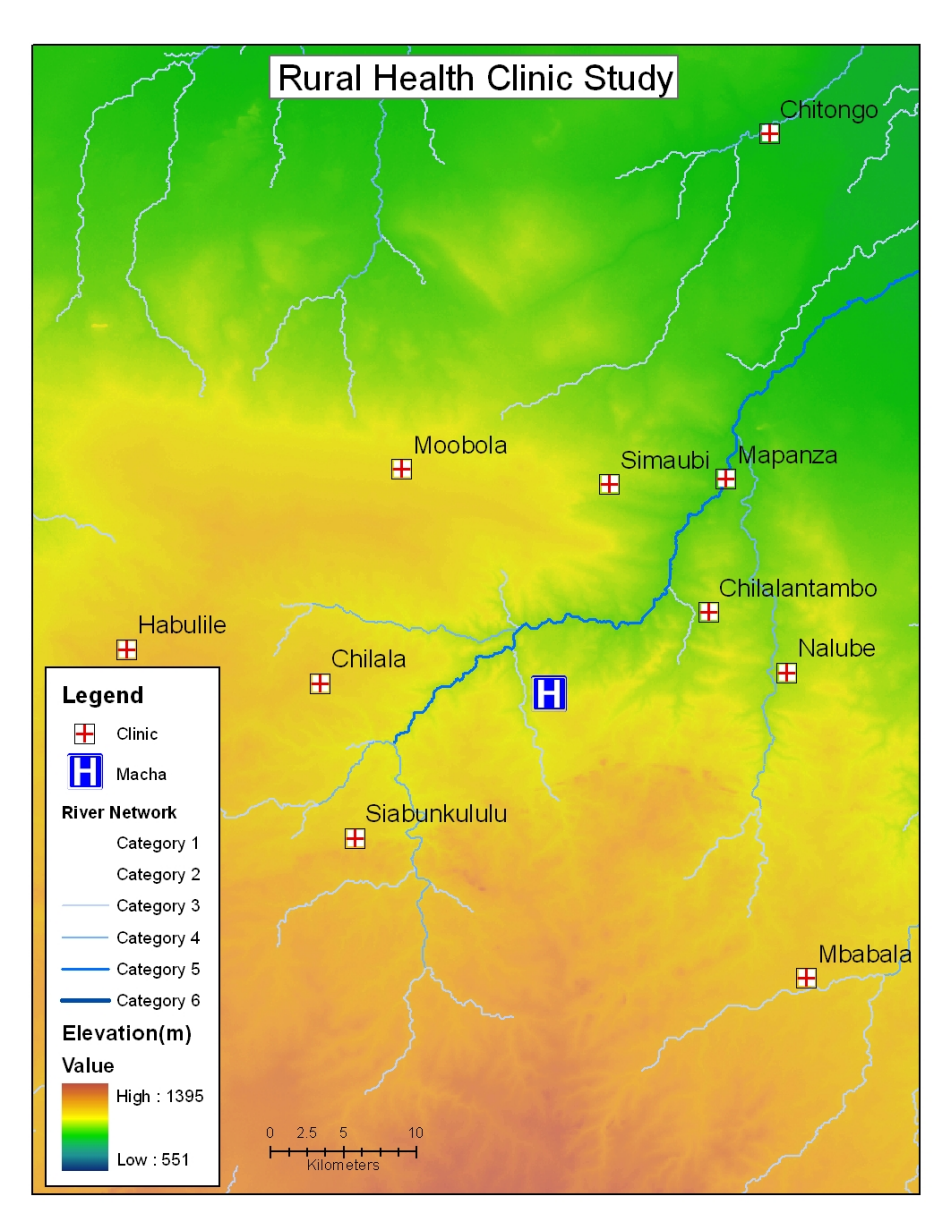
**Elevation/Contour map of the Rural Health Centre Study Area in Choma/Namwala Districts, Southern Province, Zambia**. Rural Health Centres collaborating are names and indicated. Drainage lines and river systems are indicated in categories. Category 1 is a simple drainage line that flows during and shortly after rain, Category 6 is a permanent large river.

Zambia, like so many other developing nations has recently benefited from an expanding mobile telephone service. In the area serviced by the Macha Mission Hospital (see Figure 1) this facility has come on line in the past two years and now most people in this area have access to the system and the availability of the phones is widespread. At the outset of this study (early 2008), all RHC in the approximately 5,000 Km^2 ^area of northern Choma and Namwala districts were visited and it was established that all but two centres had good reception. The area selected for the work is in the Southern Province of Zambia in the Choma and Namwala districts. The location of the various RHC participants is indicated in Figure 1 as are the main waterways classified according to permanence, drainage capacity and water flow. These are classified as "Category 1" (merely a drainage slope) to "Category 6" (major river) and indicated as such in the Figure. Information was gathered at the Malaria Institute at Macha (MIAM) located centrally. After advising appropriate authorities in the Ministry of Health and local administrations, permission was obtained to visit all rural health centres in the area. Senior staff was interviewed, the proposal explained and all personnel agreed to collaborate. The data to be collected were based entirely on malaria diagnoses done using a rapid diagnostic kit (ICT^®^) (IC Diagnostics, Cape Town) approved and supplied by the Zambia Ministry of Health. The diagnostic protocol was based on instructions from the National Malaria Control Centre (NMCC) and the Ministry of Health that required the clinical personnel to test only attendees that were currently febrile and exhibited signs indicating malaria. All positive patients were treated at no cost with artemisinin combination therapy (ACT) or sulphadoxine-pyrimethamine (SP), as recommended by the Ministry of Health. Current practice in Zambia is to use ACT as the first line treatment, with PS offered for pregnant women. The various procedures and results were entered daily in the centre's registry book. Once weekly (usually on Monday) the number of positive diagnoses and total number of RDT used per week was sent by SMS text message to one of us (AK) at MIAM central from each health centre. Actual information transmitted included the RHC name, name of the transmitting nurse, number of RDT used during the week and number of positive diagnoses with RDT. The data were entered into a spread sheet, and following checking for anomalies were transmitted from MIAM to the District Health offices as well as NMCC and Johns Hopkins personnel in Baltimore. Checking was carried out periodically by returning to each health centre and checking the SMS results against the clinic register. All attendances and diagnoses are recorded in the register on a day-to-day basis and this was used to verify previously sent information. Any discrepancies were investigated and followed up. In all health centres participating, the test sachets are retained and available for checking, even though the reading was no longer valid. If a test sachet was present as evidence the test was done, the reported result was accepted, if there was no sachet, the result was deleted from the data base.

Participating personnel were compensated for use of telephone time to cover costs and a modest stipend. Each health centre was located by GPS and its position accurately plotted on a map, which shows elevation and other features. Maps used were based on Landsat image manipulated by GIS core personnel.

### Data collection

All data were recorded weekly and entered into an Excel file system stratified by health centre. Initially 14 rural health centres were recruited into the project, however in two cases problems arose due to difficult phone access and were omitted from the study. The centres were stratified into two separate ecological zones, Kafue Flats (in the vicinity of a major flood plain) and Macha Heartland (a zone of increasing aridity and elevation). The map (Figure 1) shows the contour depiction used in this analysis. It should be mentioned that although the actual elevation range across the area is modest, the maximum difference between centres is some 200 m, the main difference between the zones is in the depth of the water table and associated water dependent ecology. In normal seasons, the water table in the Kafue flood plain 2-5 m below the ground; in the heartland, this is deeper, some 10-40 m. This reflects on the nocturnal humidity as well as the permanence of surface water in the river systems as seen in Figure 1.

Although there is no accurate estimate of current population in the research area, a census was carried out in 2001 and the results (Zambia Census 2003) are used, adjusted annually for normal population increase. The factor for this adjustment is decided by the Bureau of Statistics and published annually. Each RHC has a numerical estimate of the population served that is currently updated from the 2001 census. This information was used as the denominator to calculate the malaria incidence rate for each RHC. This denominator was chosen because it is constant over time and reflects the situation in a stable population. Data are expressed as the incidence of diagnosed malaria cases per week for each reporting health centre. There were some lapses in data collection due to occasional stock depletion or staff shortages but the process worked well most of the time, and through periodic visits to the centres, it was possible to validate the data reported by examination of the clinic attendance register. Strong interest in the work by the staff was stimulated by regular feedback from the project personnel and their occasional visits.

## Results

The results are presented as a case study to demonstrate the effectiveness of the system and the nature of malariometric information that can be acquired from timely reporting by the rural health centres in Zambia. These data demonstrate the dynamic relationship between local communities and the malaria parasites circulating in the area even where a national malaria control intervention is under way. Information presented covers two aspects of this work, first data gathering and plausibility of the system. Second, epidemiological data can be collected in real time and help improve malaria case surveillance and add to the efficiency with which resources can be used to sustain malaria control operations.

**Figure 2:**
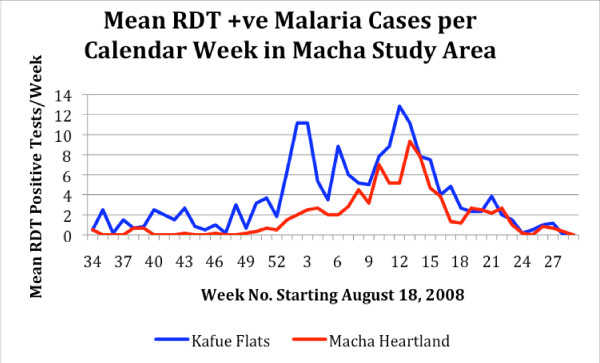
**Graph showing the mean number of malaria cases per week diagnosed using malaria rapid diagnostic tests in a series of rural health centres in a section of the Choma and Namwala Districts in the Southern Province of Zambia**. *The centres were segregated in two sections based on locality and elevation, as being in the "Kafue Flats" area (in, or close to the flood plain with elevation mainly below 1,100 m) and "Macha Heartland" (in slightly higher and drier area mainly above 1,100 m).

**Figure 3:**
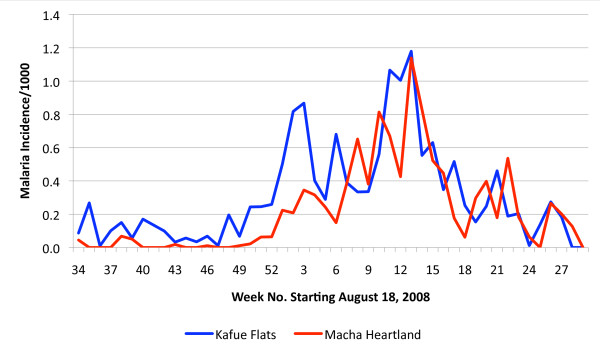
**Graph showing the incidence of malaria cases per week diagnosed using malaria rapid diagnostic tests in a series of rural health centres in a section of the Choma and Namwala Districts in the Southern Province of Zambia**.

Data collection commenced during the week 18-24 August 2008 (calendar week 34) and results expressed in Figure 2 as the mean number of cases reported weekly from all 12 health centres from week 34 until week 29 (2- 8 Mar 2009). The analysis in Figure 3 shows the weekly incidence rates (positive cases reported by the health centre per 1,000 population per week). Weekly incidence was used rather than test positivity rate (number of positive RDT per number of RDT used per week) because of the variability of use patterns by the various RHC attendants (see Table 1). During the period 18 August to 30 November very few cases of malaria were recorded in the heartland centres, whereas malaria was consistently seen mainly from the Chitongo centre, but over time expanding into Mapanza and other centres drawing patients from the lower altitudes. Following a rise in cases in the Kafue Flats around the end of December, malaria cases expanded into the heartland area with a 3-4 week delay. The time progression of case incidence is well illustrated when data are stratified by altitude and proximity to the Kafue flood plain (see Figure 1). In the heartland, malaria cases appeared only in late December while in the flood plain area cases occurred throughout the study period and peaked some 8-10 weeks before the disease expanded in the overall area. It should be noted that the cases occurred in spite of an ongoing anti-malaria intervention using insecticide treated bed nets. These nets were distributed by the National Malaria Control Centre to achieve at least one net per sleeping hut throughout the area, including the flood plain area in June 2008.

**Table 1:** Showing use patterns of Malaria Rapid Diagnostic tests made at 12 Rural Health Centres in the South Province of Zambia.

				Weekly Mean			
						
Health Centre	Elevation (m)	No. Weeks	No. Weeks with RDT Stock Outs	Total +ve (95% CI)	Total Tests (95% CI)	Proportion (%) Tests Positive (no. +ve/no. tests)	Total Population (Census Data 2003)
Chitongo	1013	48	3	10.39 (7.70-13.08)	123.07 (107.0-139.14)	9.03	14,666

Mapanza	1059	47	1	7.92 (6.36-9.48)	62.15 (51.55-72.75)	10.53	17,405

Chilalantambo	1100	48	12	0.90 (0.49-1.32)	3.56 (2.49-4.63)	33.59	2,614

Moobola	1165	44	8	1.68 (0.97-2.39)	55.91 (46.33-65.49)	3.78	14,356

Simaubi	1117	44	8	2.36 (1.20-3.52)	26.62 (21.84-31.40)	11.49	7,506

Mangunza	1087	36	0	1.31 (0.50-2.12)	10.6 (8.20-13.01)	12.67	9,756

Chilala	1187	47	2	1.60 (0.87-2.33)	10.77 (8.69-12.85)	15.82	9,630

Habulile	1210	48	0	3.58 (2.14-5.02)	23.40 (11.30-35.49)	15.64	7,223

Macha	1083	48	0	2.42 (1.48-3.35)	35.71 (28.87-42.54)	6.77	15,290

Mbabala	1204	37	0	0.27 (0.01-0.52)	26.83 (22.04-31.36)	1.04	9,981

Nalube	1110	47	3	0.64 (0.30-0.97)	7.45 (6.14-8.75)	9.15	2,614

Siabunkulu	1190	38	0	2.89 (1.29-4.48)	21.74 (14.23-29.24)	13.33	9,246

For orientation see Map Figure 1.

The data in Table 1 are based on the use pattern of rapid diagnostic tests administered in 12 rural health centres in the study area and show mean number of positive diagnosis and the proportion of tests that were positive made per week for the period of observation in each of the health centres. There was considerable variation in the use pattern of the health workers, although one centre (Chilalantambo) had numerous stock outs over the year and personnel at this centre probably were more selective in the use of the test than in most of the other centres. The table also shows the total number of people who reside in the region supported by the health centre as reported by the last national census. The proportion of positive results compared to total tests administered is a crude estimate of treatments saved, because according to instructions each test was administered to a case that clinically would have been considered clinical malaria and treated accordingly.

## Discussion

The focal nature of malaria infection constitutes a challenge to agencies charged to control the disease in a sustainable manner. Because of the seasonal ebb and flow of cases, it would be strategic to identify foci at a time when transmission is restricted, as this is the time when the parasite population is amenable to elimination. Recent work by Shekalaghe *et al *[7] as well as Killeen *et al *[8] in areas of low parasite transmission has shed light on the importance, as well as the location, of populations with silent infections of malaria [9] as a reservoir source of parasite gametocytes.

Because of a lack of sensitive detection methods, past control programmes that were unable to detect the occurrence of reservoirs of infection were prone to a rapid return to high endemicity when the intervention foundered. The key would be to utilise information that is currently available through the normal health system to help identify such foci.

In this study, an objective was to demonstrate the importance of local RHC facilities and their expertise to locate these parasite reservoirs. Another objective was to demonstrate the role the RHC can play in timely case detection. Local health system personnel were seen as a resource in facilitating active case detection and with the current infrastructure available noting they could play an important role in an effective malaria surveillance system that can transmit critical information in a timely manner. The information can help to detect index cases of malaria during the low transmission period prior to the usual seasonal spread of malaria into areas where transmission is unstable and where it impacts so severely in the human population.

Data of epidemiological importance were also collected in this study. With the ability to follow the incidence of diagnosed malaria in a time and spatially distinct manner, it would be possible to map the location of health centres with highest incidence of malaria. Stratification of the centres by location showed a marked difference in malaria incidence when comparing those in the zone of the Kafue Flats and the others located in the Macha "heartland". Certainly it appeared that malaria persisted at a low level in the Kafue Flats zone for over 10 weeks during the time the weather was hot and dry after which an upsurge in cases was seen following the advent of the rainy season. This upsurge occurred some four weeks prior to a similar situation in the "heartland", and corresponded with onset of a stable rainy period. This information can provide the health services a window of time to mount a limited intervention at a time when the reservoir is vulnerable. Such a targeted intervention either with vector control or anti-gametocyte chemotherapy could have a major impact on restructuring a national programme.

From Table 1 it is clear that the use of the RDT was not consistent. In Zambia, rural health authorities are instructed to test only patients that present with fever and other symptoms that might suggest malaria. Considering the role of the RDT for diagnoses at the clinical level, there was an increase in the use of this test and a significant reduction in the use of anti-malarial drugs, about 90% reduction if all "clinical" cases had been actually treated without the confirmation of the test. Table 1 shows the inconsistency in the proportion of tests used compared to positive diagnoses in the various health centres. Perhaps if the symptoms were defined more specifically, the use patterns of the various centres might be might be more consistent but clinical malaria is difficult to define and other studies have shown this discrepancy [10]. What is important, however, is that a positive RDT diagnosis is a piece of real data. It represents a real case within the limits of sensitivity and specificity of the particular product and as such it is of epidemiological importance. Such information has not been readily available, but now it can be seen to be highly relevant to health authorities and planners of effective interventions.

Use patterns of the diagnostic test affect the overall cost effectiveness of the RDT, however, one needs to take into consideration the experience and training of the personnel that work at the grass root level. Previously with less expensive drugs there was emphasis to treat any febrile case as malaria, because it was curable and also a frequent cause of death. Staff remember this but now instructions, countermand past experience. Bringing an objective test into the rural health system where the onus of diagnosis is in the hands of a single person may take some orientation. It is important to appreciate that some workers may feel the test is less reliable than their knowledge and experience. Use of the test will vary with personalities until there is a high level of confidence among the RHC staff. In the long run, as the RDT gains acceptance, and use of the drugs is restricted, considerable savings will result from a reduction of the number of tests administered.

## Conclusions

The network of rural health workers recruited into this work was shown to be receptive, interested and highly cooperative over the year of operation. This level of participation was continually reinforced by regular feedback of data, information and encouragement by the MIAM staff that also occasionally assisted with supplies when these were short. The method of communication through mobile phone system was effective and inexpensive. All expenses and reimbursements were handled through the SMS system. Modern communication facilities now exist over most of Africa and could easily be incorporated into regular, time sensitive data retrieval systems that could serve government health systems as an important arm of any health related programme. Enrolling rural health centres to provide timely information on the occurrence of malaria cases to central health authorities will provide critical data enabling rapid deployment of specific interventions. In the case described in this paper, timely information will improve management of the national malaria control programmes and could enable targeted deployment of specific drug intervention to prevent transmission of malaria and reduce the expansion of the disease into a previously controlled area.

## Competing interests

The authors declare that they have no competing interests.

## Ethical approval

The study was performed with approval from the University of Zambia Research Ethics Committee 004-01-07, and the Johns Hopkins School of Public Health Ethical Review Board IRB number 00000229.

## Authors' contributions

AK was overall coordinator and managed the work, PM performed liaison with the health centre staff, GS performed analyses, SM was overall coordinator in Zambia and CS set up the project, obtained funding and wrote the paper. All authors read and approved the manuscript.
